# Corticosteroids or NSAIDs in Managing Acute Respiratory Infections: Valuable Differences

**DOI:** 10.1155/jimr/9991040

**Published:** 2026-05-12

**Authors:** Francesco Scaglione, Giorgio Ciprandi

**Affiliations:** ^1^ Department of Oncology and Hemato-Oncology, Postgraduate School of Clinical Pharmacology and Toxicology, University of Milan, Milan, Italy, unimi.it; ^2^ Department of Medicine and Health Sciences, University of Molise, Campobasso, Italy, unimol.it

**Keywords:** acute respiratory infections, corticosteroids, immunity, inflammation, nonsteroidal anti-inflammatory drugs, oxidative stress

## Abstract

Nonsteroidal anti‐inflammatory drugs (NSAIDs) are commonly used for respiratory infections. These drugs work by blocking two enzymes, COX‐1 and COX‐2, which regulate the production of prostaglandins, mediators involved in pain, fever, and inflammation. Corticosteroids (CSs) are commonly used in outpatient settings for anti‐inflammatory purposes in the treatment of infectious diseases. However, their potential side effects, such as immunosuppression and increased metabolism, can be overlooked, even at low doses. Their use is clearly defined for severe conditions. Other infections, such as community‐acquired pneumonia, pharyngotonsillitis, or otitis, do not have supportive data for the use of systemic CSs. For COVID‐19, CSs are beneficial for severe cases that require ventilation, while they may not be helpful in mild cases. Infection‐induced inflammation is associated with oxidative stress, a condition that arises from an imbalance between reactive oxygen species (ROS) and inadequate antioxidant responses. This stress worsens inflammatory reactions and can lead to severe respiratory infections and tissue damage. Managing oxidative stress is crucial in treating respiratory infections, and both CSs and NSAIDs can help reduce it. NSAIDs are preferred for treating symptoms such as fever and pain during the early phases of infections, especially viral ones, without hindering the immune response. CSs are powerful anti‐inflammatory medications that are useful for treating infections in patients with asthma or allergies. However, CSs are not recommended for relieving pain and fever and can weaken the immune response. Both NSAIDs and steroids can mask serious infections, so doctors must be cautious in their use.

## 1. Introduction

Worldwide, respiratory tract infections (RTIs) are the leading cause of death in childhood and the fourth most common cause of death in adults [1]. So, RTIs represent a global health concern with the greatest risk for young children, older people, and immunocompromised patients [2].

The clinical spectrum ranges from mild infection to severe or fatal disease, and the severity depends on the interaction among the causative agent, environmental conditions, and host immune response capacity [3]. Respiratory infections typically occur as acute diseases with a rapid clinical onset, ranging from hours to days after infection.

The inflammatory response occupies a central position in the pathophysiology of infectious diseases, representing both the primary host defense mechanism and the source of the most distressing clinical symptoms. Acute inflammation typically manifests as pain, fever, local swelling, and functional impairment, symptoms that, while biologically purposeful, significantly impact patient quality of life [4]. For this reason, anti‐inflammatory drugs from various classes have a long history of use in managing patients with respiratory infections to relieve annoying symptoms, such as pain and fever.

Beyond symptomatic relief, anti‐inflammatory agents can modulate the host immune response, potentially limiting the tissue damage associated with excessive or dysregulated inflammation, a mechanism of particular relevance in severe infections, such as influenza and COVID‐19 [5]. The present publication aims to discuss the use of corticosteroids (CSs) and nonsteroidal anti‐inflammatory drugs (NSAIDs) in the treatment of infections, as well as the associated risks, based on published findings in this area.

### 1.1. NSAIDs

The drugs in this group, most commonly used for respiratory infections, include ketoprofen lysine salt (KLS), ibuprofen, ibuprofen lysine salt, diclofenac, naproxen, and acetylsalicylic acid. Their pharmacological activity relies on the dual inhibition of COX‐1 and COX‐2, the enzymatic isoforms that catalyze the conversion of arachidonic acid into prostanoids. This heterogeneous lipid mediator family, encompassing prostaglandins (PGE2, PGD2, PGF2α), prostacyclin (PGI2), and thromboxane A2, exerts pleiotropic effects across the hematological, pulmonary, renal, and cardiovascular systems, underpinning the cardinal symptoms of respiratory infections [6–9].

Many mediators synthesized by COX‐1 and COX‐2 are strongly implicated in immune reactions following infections. Among prostanoids, PGE2 and PGI2 are the principal mediators of the pro‐inflammatory response: they sensitize nociceptors, induce pyrexia, and increase microvascular permeability, thereby promoting tissue edema and leukocyte extravasation [6, 7]. PGD2, predominantly generated by mast cell COX activity, amplifies the allergic inflammatory cascade [8]. Thromboxane A2, in turn, is a potent activator of platelet aggregation and vasoconstriction, contributing to thrombotic complications [9].

Moreover, COX1 and COX2 have crucial effects on the host immune response to both bacterial and viral infection. In particular, among the prostanoids produced by COX, PGE2 plays a critical role by regulating the expression levels of many serum proteins, playing an essential role in systemic inflammation and virus‐induced alveolar and interstitial pneumonia [10, 11].

Experimental evidence indicates that PGE2 amplifies NF‐κB‐driven cytokine production; crucially, pharmacological reduction of PGE2 levels does not impair host antiviral defenses and may in fact facilitate viral clearance [12, 13].

Therefore, NSAIDs, by inhibiting COX‐2 activity, reduce PGE2 synthesis, offering obvious benefits in the progression of infectious diseases. Through the inhibition of COX isoforms, NSAIDs modulate the biosynthesis of pro‐inflammatory prostanoids and downstream cytokine signaling, thereby attenuating the hyperinflammatory cascade, including hyper‐inflammation and cytokine storm, that characterizes severe viral respiratory infections [14, 15].

**Figure 1:**
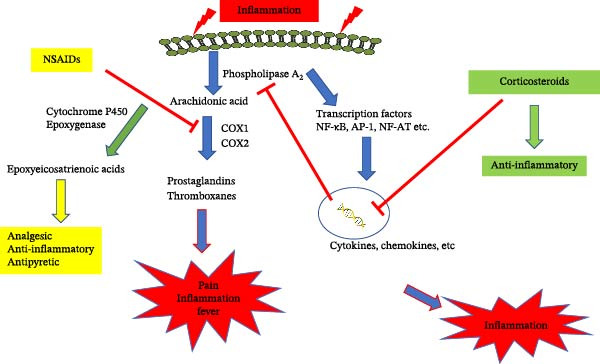
Main anti‐inflammatory mechanisms of action of NSAIDs and corticosteroids. NSAIDs, by inhibiting COX1 and COX2, reduce the production of proinflammatory prostaglandins, consequently increasing the substrate (arachidonic acid) for cytochrome P450, increasing the synthesis of epoxyeicosatrienoic acids (EETs) with endogenous anti‐inflammatory and analgesic activity. Corticosteroids, although having a strong anti‐inflammatory activity, do not have direct analgesic and antipyretic activity.

Interestingly, COX inhibition increases the levels of epoxyeicosatrienoic acids (EETs) because arachidonic acid, the common substrate for both the COX and cytochrome P450 (CYP) pathways, is shunted away from the blocked COX enzyme and toward the CYP450 epoxygenase pathway. This redirection of substrate increases the production of EETs, which are anti‐inflammatory, vasodilatory, and analgesic mediators (Figure 1) [16, 17].

COX inhibition by NSAIDs leads to a reduction in inflammatory symptoms such as fever and pain. Furthermore, NSAIDs have been shown to inhibit disease progression to complications and hospitalizations when used early in the course of infection [18, 19].

NSAIDs are typically divided into groups based on their chemical structure, as reported in Table 1.

**Table 1:** Most commonly used NSAIDs according to chemical structure, relative risk (RR) for gastrointestinal (GI) and cardiovascular (CV) adverse effects.

Drugs	GI damage	CV damage
Nonusers	1	1
Heteroaryl acetic acid		
Ibuprofen	1.8	1.25
Ketoprofen acid	3.9	1
Ketoprofen lysine salt	1.5	1
Naproxen	4.1	1.22
Anthranil acid		
Diclofenac	3.3	1.28
Ketorolac	11.5	1.8
Indol and indene acetic acid		
Indomethacin	4.1	1.51
Salicylic acid derivates		
Acetylsalicylic acid (high dose)	3.8	1
Ortho‐diarylheterocycles (coxibs)		
Celecoxib	1.5	1.15
Etoricoxib	2.3	1.39

The efficacy of NSAIDs is comparable among various molecules when administered at equivalent doses; however, tolerability can vary significantly depending on the molecule used.

Here, we will focus on the negative impact of NSAIDs on the gastrointestinal and cardiovascular systems, which, in our opinion, can impact the majority of the population.

The development of gastric‐duodenal mucosal lesions has been the main limitation when NSAIDs were used long‐term in the treatment of chronic inflammatory conditions. A prevalence of gastric mucosal lesions has been observed in patients with rheumatoid arthritis or pain who took NSAIDs [20, 21].

The traditional view attributing NSAID‐induced gastric injury solely to COX‐1‐mediated suppression of gastroprotective PGE2 and PGI2 has been progressively challenged. Accumulating evidence demonstrates that COX enzymes also participate in mucosal repair and ulcer resolution, suggesting a more complex, bidirectional role in gastric homeostasis [22, 23]. A large‐scale meta‐analysis by the CNT Collaboration, pooling data from over 124,000 participants, confirmed that all major NSAIDs—including ibuprofen, diclofenac, naproxen, and selective COX‐2 inhibitors—are associated with a significantly elevated risk of upper gastrointestinal complications [24]. Over the last 20 years, a series of studies has demonstrated that the COX‐1 pathway is not the only pathway underlying NSAID toxicity in the gastric mucosa. As weak organic acids, NSAIDs interact directly with the phospholipid bilayer of the gastroduodenal mucosa, reducing its surface hydrophobicity. This physicochemical disruption compromises the protective mucosal barrier, rendering the epithelium vulnerable to luminal noxious agents, including gastric acid, pepsin, bile salts, and enteric bacteria [25–27]. Furthermore, a growing body of experimental evidence demonstrates that NSAIDs trigger reactive oxygen species (ROS) overproduction in the gastric mucosa, leading to mitochondrial dysfunction, bioenergetic crisis, and activation of apoptotic pathways—collectively referred to as mitochondrial oxidative stress (MOS) [24, 28].

Homeostasis of the gastric environment is maintained by balancing protective factors, such as mucus, against aggressive factors, including gastric acid and stress. An increase in aggressive factors or a decrease in protective factors can lead to gastric damage [29].

Beyond COX inhibition, direct physicochemical interactions between NSAIDs and the gastric mucosa represent an important, often underappreciated source of gastrointestinal toxicity. In this context, lysine salification of ketoprofen or ibuprofen has been shown to substantially improve gastric tolerability, both by neutralizing the local acidic effect of the parent molecule and by upregulating mucosal cytoprotective mechanisms [30–34].

Both selective and nonselective COX2 inhibitors were associated with an increased risk of serious ischemic vascular events, predominantly myocardial infarction [33, 34].

These findings suggest that different, non‐COX‐mediated mechanisms may be involved in cardiovascular toxicity. Mechanistic studies have revealed that diclofenac, unlike ketoprofen, exerts direct cardiotoxic effects despite comparable COX2/COX1 selectivity. This toxicity appears to be mediated by mitochondrial membrane depolarization and consequent proteasomal dysfunction, ultimately triggering cardiomyocyte apoptosis [35].

The structural heterogeneity of NSAIDs underlies their pharmacokinetic and tolerability differences. Based on their chemical scaffold, they are conventionally grouped into the following classes (Table 1), each associated with distinct risk profiles.

Among the NSAIDs with the highest gastrointestinal risk are ketorolac, naproxen, and indomethacin, while those with the highest cardiovascular risk are ketorolac, indomethacin, etoricoxib, and diclofenac. Among the most commonly used drugs, ketoprofen appears to be the one with the lowest cardiovascular risk [36].

NSAIDs can also cause adverse renal effects. NSAID‐mediated suppression of renal prostaglandin synthesis impairs the vasodilatory tone that normally maintains glomerular filtration rate and renal perfusion. This mechanism is clinically relevant primarily in patients with pre‐existing hemodynamic compromise, such as those with heart failure, volume depletion, or chronic kidney disease—who depend on prostanoid‐mediated afferent arteriolar dilation to preserve renal function. In subjects with intact renal reserve, short‐term NSAID exposure carries a substantially lower nephrotoxic risk [37].

### 1.2. CSs

Glucocorticoids (GCs) are a heterogeneous group of drugs with similar mechanisms of action but very different pharmacodynamic characteristics (Table 2).

**Table 2:** Properties, dosing equivalents of systemic corticosteroids, relative to hydrocortisone.

Drugs	Equivalent dose (mg)	Anti‐inflam. potency^a^	Salt retaining^a^	HPAA suppressive potency^a^	Duration of action (h)
Hydrocortisone	20	1	1	1	8–12
Cortisone	25	0.8	0.8	1	8–12
Deflazacort	6–7.5	3	0	1–2	8–12
Prednisolone	5	4	0.8	1	12–36
Prednisone	5	4	0.8	1	12–36
Methylprednisolone	4	4–5	0.2–0.5	5	12–36
Triamcinolone	4	5–10	0	5	12–36
Dexamethasone	0.75	25	0	50	36–72
Betamethasone	0.75	25	0	50	36–72

Abbreviation: HPA, hypothalamus‐pituitary‐adrenal axis.

^a^Relative to hydrocortisone.

CSs exert their anti‐inflammatory effects by influencing multiple signal transduction pathways.

At the molecular level, CSs exert their effects primarily through binding to the cytoplasmic GC receptor (GR), forming a ligand‐receptor complex that translocates to the nucleus. Here, it modulates gene transcription both directly, via GC‐responsive elements (GREs), and indirectly, through interference with pro‐inflammatory transcription factors such as NF‐κB and AP‐1, resulting in broad suppression of cytokine and chemokine gene expression [38].

Furthermore, CSs can activate several anti‐inflammatory genes and increase the degradation of mRNA encoding specific inflammatory proteins. In particular, GCs induce GC‐induced leucine zipper and Annexin A1, proteins intrinsically involved in anti‐inflammatory functions [38]. This broad range of actions explains the effectiveness of CSs in complex inflammatory diseases, such as asthma and rheumatoid arthritis.

CSs, for anti‐inflammatory purposes in infectious diseases, are widely used in outpatient medical practice. Unfortunately, alongside their positive effects on specific symptoms, the risks associated with their other properties (immunosuppression and hypermetabolism) are often overlooked, even when administered at low doses for short‐term use [39, 40].

Scientific societies recommend local intranasal application of CSs and do not consider systemic CS therapy [41, 42].

Expert groups and position papers recommend the use of CSs in local applications for moderate to severe rhinosinusitis, emphasizing that the addition of systemic CSs therapy shows clear benefits only in cases of chronic rhinosinusitis with polyposis [43, 44].

Regarding other infections, such as pharyngotonsillitis or acute otitis media, the routine use of systemic CSs is not recommended [45, 46].

GCs are also used to reduce the symptoms and consequences of serious infections. In bacterial meningitis, adjunctive GC therapy has been shown to attenuate the inflammatory cascade responsible for secondary neurological injury, including cerebral edema, raised intracranial pressure, and vasculitis. However, their benefit is limited to preventing further damage, they cannot reverse neurological deficits already established at the time of treatment initiation [47].

CSs are generally not indicated in CAP (e.g., IDSA/ATS guidelines do not recommend CS in CAP), except when associated with COPD, asthma, or an autoimmune disease [48]. Similarly, the Surviving Sepsis Campaign guidelines recommend the use of hydrocortisone in CAP patients who present with septic shock refractory to fluid resuscitation and vasopressor support. In patients with influenza, CS have not shown any benefit and may actually increase the risk of complications, such as invasive pulmonary aspergillosis [49]. Furthermore, the use of systemic CSs is not routinely recommended in preschool children with acute wheezing attacks who do not have asthma or allergies [50, 51]. In patients with COVID‐19, there is a clear benefit to the use of CSs in those patients who have severe disease and require ventilation. In patients with mild or early disease, the use of CSs may be counterproductive [52]. While the benefit of CSs in mechanically ventilated patients seems clear, many points remain a source of debate. Excessively long treatment durations appear to increase mortality, while the association with immunosuppressants (e.g., tocilizumab) appears favorable. Finally, it seems that despite the improvement, the long‐term prognosis does not change [53–55].

In addition, it has to be underlined that infection‐induced inflammation is typically associated with oxidative stress [56]. Respiratory infections trigger a state of oxidative imbalance, in which ROS generation outpaces endogenous antioxidant capacity. This redox dysregulation amplifies the local inflammatory response and contributes to tissue injury, particularly in severe disease phenotypes [57]. Thus, contrasting oxidative stress constitutes a cornerstone in the treatment of respiratory infections. In this regard, there is evidence that both CSs and NSAIDs may mitigate oxidative stress [58, 59].

## 2. Conclusions

Anti‐inflammatory drugs are frequently used in conjunction with infections to relieve associated symptoms, such as fever and pain. In the early management of viral respiratory infections, NSAIDs represent a rational therapeutic choice: their mechanism of action targets the prostanoid pathway, providing symptomatic relief while preserving, and in some cases enhancing, the host’s antiviral immune response [5]. CSs exert broad anti‐inflammatory effects through genomic mechanisms but lack direct analgesic and antipyretic activity, making them less suitable than NSAIDs for symptomatic relief of fever and pain. Furthermore, their capacity to suppress cellular immunity, including lymphocyte and macrophage function, distinguishes them mechanistically from NSAIDs, which do not directly impair inflammatory cell viability. However, they play an essential role in treating inflammation caused by infections in selected groups, such as those with advanced COVID‐19.

While we will not address the extensive topic of drug interactions here, it is nevertheless important to briefly consider potential drug interactions when choosing both NSAIDs and CSs.

Clinically relevant pharmacodynamic and pharmacokinetic interactions have been documented between NSAIDs and several commonly coprescribed drug classes, including antiplatelet agents, antihypertensives, and serotonergic antidepressants. The magnitude of these interactions is generally exposure‐dependent; therefore, a careful assessment of NSAID type, dosage, treatment duration, and concomitant medications is essential to minimize the risk of adverse outcomes [60].

CSs, particularly when taken orally, have significant interactions with medications such as anticoagulants (warfarin), NSAIDs (ibuprofen), diabetes medications, and some HIV medications, increasing the risk of bleeding and gastrointestinal ulcers and reducing the effectiveness of treatment. They can also cause electrolyte imbalances (low potassium levels) and interact with antifungals and anticonvulsants [61]. It should be remembered that they also reduce the patient’s immune response. Furthermore, they can cause systemic adverse effects, such as osteoporosis and diabetes.

A final consideration applicable to both drug classes is the risk of symptom masking: effective suppression of fever and inflammatory signs may obscure the clinical evolution of a serious or worsening infection, potentially delaying appropriate diagnosis and management. Clinicians should therefore maintain a high index of suspicion, particularly in vulnerable patient populations.

## Funding

No funding was received for this manuscript.

## Conflicts of Interest

The authors declare no conflicts of interest.

## Data Availability

The data that support the findings of this study are available from the corresponding author upon reasonable request.
